# Cereblon-Based Small-Molecule Compounds to Control Neural Stem Cell Proliferation in Regenerative Medicine

**DOI:** 10.3389/fcell.2021.629326

**Published:** 2021-03-11

**Authors:** Tomomi Sato, Takumi Ito, Hiroshi Handa

**Affiliations:** ^1^Department of Chemical Biology, Tokyo Medical University, Tokyo, Japan; ^2^Department of Anatomy, School of Medicine, Saitama Medical University, Saitama, Japan; ^3^Department of Obstetrics and Gynecology, School of Medicine, Saitama Medical University, Saitama, Japan

**Keywords:** zebrafish, thalidomide, cereblon, neural stem cells, brain development, CELMoDs, PROTAC

## Abstract

Thalidomide, a sedative drug that was once excluded from the market owing to its teratogenic properties, was later found to be effective in treating multiple myeloma. We had previously demonstrated that cereblon (CRBN) is the target of thalidomide embryopathy and acts as a substrate receptor for the E3 ubiquitin ligase complex, Cullin-Ring ligase 4 (CRL4^CRBN^) in zebrafish and chicks. *CRBN* was originally identified as a gene responsible for mild intellectual disability in humans. Fetuses exposed to thalidomide in early pregnancy were at risk of neurodevelopmental disorders such as autism, suggesting that CRBN is involved in prenatal brain development. Recently, we found that CRBN controls the proliferation of neural stem cells in the developing zebrafish brain, leading to changes in brain size. Our findings imply that CRBN is involved in neural stem cell growth in humans. Accumulating evidence shows that CRBN is essential not only for the teratogenic effects but also for the therapeutic effects of thalidomide. This review summarizes recent progress in thalidomide and CRBN research, focusing on the teratogenic and therapeutic effects. Investigation of the molecular mechanisms underlying the therapeutic effects of thalidomide and its derivatives, CRBN E3 ligase modulators (CELMoDs), reveals that these modulators provide CRBN the ability to recognize neosubstrates depending on their structure. Understanding the therapeutic effects leads to the development of a novel technology called CRBN-based proteolysis-targeting chimeras (PROTACs) for target protein knockdown. These studies raise the possibility that CRBN-based small-molecule compounds regulating the proliferation of neural stem cells may be developed for application in regenerative medicine.

## Introduction

Thalidomide—originally developed as a sedative-hypnotic drug and used worldwide approximately 60 years ago—is an effective antiemetic and prescribed for morning sickness during pregnancy. This drug was withdrawn from the market because newborns showed multiple birth defects when pregnant women consumed the drug during early pregnancy. Thalidomide is associated with a range of teratogenicity, termed thalidomide embryopathy, in the ears, eyes, face, limbs, genitalia, and internal organs, including heart, kidney, and gastrointestinal tract (Miller and Strömland, 1999; Vargesson, 2015). Thalidomide teratogenicity shows different critical exposure periods during embryogenesis. Previous research indicates that the earliest exposure to thalidomide increases the risk of autism and epilepsy (Strömland et al., 1994; Miller and Strömland, 1999; Miller et al., 2005). Notably, limb malformations in embryos exposed to thalidomide were observed in humans and rabbits but not in rodents, implying that thalidomide teratogenicity is species-specific (Fratta et al., 1965; Schumacher et al., 1968).

We previously identified cereblon (CRBN) as a primary direct target of thalidomide (Ito et al., 2010). *CRBN*, encoding a 442-amino-acid protein, is identified as a gene responsible for autosomal recessive non-syndromic intellectual disability; a nonsense mutation, p.R419X, generates a truncated CRBN lacking 24 amino acids at the C-terminal owing to the presence of a premature stop codon (Higgins et al., 2004). Another missense mutation in *CRBN* is also associated with severe intellectual disability and seizures (Sheereen et al., 2017). CRBN serves as a substrate receptor of the Cullin-Ring ligase 4 E3 ubiquitin ligase complex (CRL4^CRBN^) that recognizes substrates for ubiquitination and subsequent proteasomal degradation (Ito et al., 2010). CRBN is identified as a protein directly interacting with the cytosolic carboxy-terminus of large conductance, Ca^2+^- and voltage-activated K^+^ (BK) channel α subunit (Jo et al., 2005). However, the significance of these mutations with respect to intellectual disability and cellular functions of CRBN is still unclear.

Although the mechanisms underlying the sedative function of thalidomide has not been elucidated, thalidomide has been found to have therapeutic effects in the context of erythema nodosum leprosum and multiple myeloma. At present, thalidomide and its derivatives lenalidomide and pomalidomide are repurposed as immunomodulatory drugs (IMiDs) for blood cancers. Accumulating evidence suggests that thalidomide and IMiDs bind to CRBN, thereby altering substrate recognition depending on the ligand structure and exerting therapeutic effects by degrading different ligand-specific substrates (neosubstrates) (Chamberlain and Cathers, 2019; Ito and Handa, 2020). Elucidation of the molecular mechanisms of action of thalidomide and IMiDs promoted the development of a new protein knockdown technique, proteolysis-targeting chimeras (PROTACs), originally developed using another E3 ubiquitin ligase, von Hippel-Lindau (Sakamoto et al., 2001; Pettersson and Crews, 2019).

We demonstrated that thalidomide caused limb defects through CRBN in chicks and zebrafish (Ito et al., 2010; Asatsuma-Okumura et al., 2019), suggesting that basic molecular mechanisms of limb development involving CRBN are evolutionarily conserved among vertebrates. Zebrafish (*Danio rerio*) is an excellent model organism to investigate molecular genetic and pathogenic mechanisms underlying human diseases that have a developmental origin (Lieschke and Currie, 2007). Each fish lays many eggs, and the transparent small embryos develop externally in a dish, enabling us to easily observe and analyze the effects on development by gene expression, knockdown, knockout, and screening of small-molecule libraries using living whole embryos (Driever et al., 1994; Kaufman et al., 2009; D’Amora and Giordani, 2018). Furthermore, many transgenic lines, mutants, and disease models are currently available for studying neurodevelopmental disorders in zebrafish (Meshalkina et al., 2018; Sakai et al., 2018; Vaz et al., 2019).

In this mini review, we summarize recent advances in our understanding of the teratogenic and therapeutic effects of thalidomide and its derivatives, currently called cereblon E3 ligase modulators (CELMoDs), and describe the potential of CELMoDs for developing small-molecule compounds that regulate neural stem cell (NSC) proliferation and their significance in regenerative medicine.

## Teratogenic Effects of Thalidomide in Humans

In human fetal development, 4–15 weeks gestation (3–14 weeks post-conception) is the organogenesis period; especially, 4–9 weeks gestation (3–8 weeks post-conception) is critical for organogenesis. Human embryos in the first 8 weeks are highly sensitive to teratogens (Wilson, 1973; Hill, 2007). The earliest exposure to thalidomide (20–24 days post-fertilization) has been reported to increase the risk of autism and epilepsy (Strömland et al., 1994; Miller and Strömland, 1999; Miller et al., 2005). Brain development initially begins with the induction of neuroectoderm from ectoderm during gastrulation. This neural induction occurs 3 weeks post-fertilization in humans, roughly corresponding to the critical period for autism elicited by thalidomide exposure (Strömland et al., 1994; Miller and Strömland, 1999; Miller et al., 2005). This suggests that thalidomide has the potential to affect early brain development when neuroectodermal cells, including NSCs, dramatically proliferate and differentiate.

**FIGURE 1:**
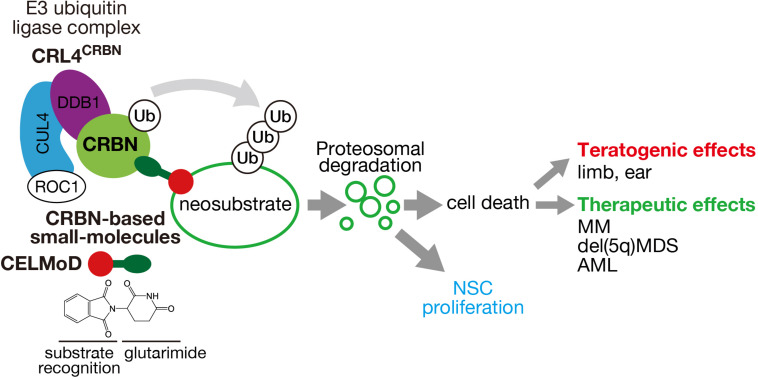
Mechanism of action of CRL4^CRBN^ E3 ubiquitin ligase and its effects through CRBN-based small molecules. CRBN, a substrate receptor of CRL4^CRBN^, binds to CRBN-based small molecules (IMiDs, CELMoDs, PROTACs) through the glutarimide moiety (green ellipse) and recognizes different neosubstrates depending on the structure (red circle). Polyubiquitination and proteasomal degradation of the target neosubstrates cause cell death or presumably NSC proliferation that leads to various teratogenic and therapeutic effects.

**FIGURE 2:**
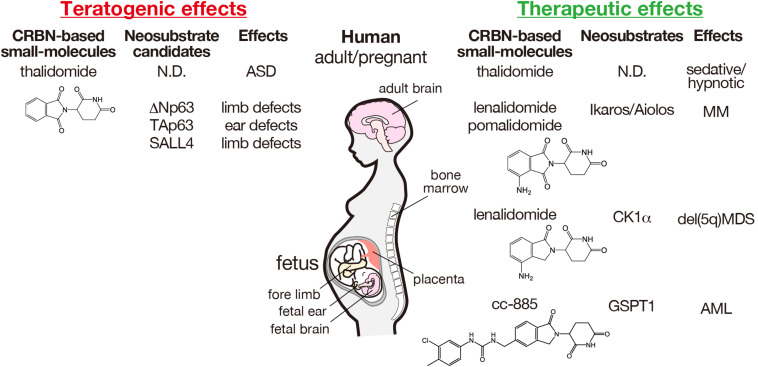
Teratogenic and therapeutic effects of CRBN-based small molecules and the corresponding neosubstrates in humans. The structures of thalidomide and its derivatives are indicated. AML, acute myeloid leukemia; ASD, autism spectrum disorder; MDS, myelodysplastic syndrome; MM, multiple myeloma; ND, not determined.

Although the precise molecular mechanisms underlying thalidomide teratogenicity remain obscure, multiple teratogenic effects were considered due to the functional CRBN inhibition, as CRBN is a direct protein target of thalidomide that inhibits the auto-ubiquitination of CRBN (Ito et al., 2010). Furthermore, thalidomide teratogenicity implies that CRBN plays a critical role in human fetal development. However, CRBN-knockout mice exhibit normal brain development, but show impaired presynaptic function owing to enhanced BK channel activity and deficits in hippocampal-dependent learning and memory via exaggerated AMP-activated protein kinase (AMPK) activity (Higgins et al., 2004; Bavley et al., 2018; Choi et al., 2018). Forebrain-specific conditional CRBN-knockout mice also show hippocampus-dependent deficits in associative learning (Rajadhyaksha et al., 2012). Although the role of CRBN in brain development remains poorly understood, investigation of the molecular mechanisms underlying the therapeutic effects of thalidomide and IMiDs revealed that CELMoDs bind to CRBN to alter substrate recognition, leading to ligand-specific neosubstrate degradation (Figure 1). Two different proteins, spalt-like transcription factor 4 (SALL4) and tumor protein 63 (TP63, p63), have been identified as CRBN neosubstrates responsible for the teratogenic effects of thalidomide in the limb and ear (Donovan et al., 2018; Matyskiela et al., 2018; Asatsuma-Okumura et al., 2019, 2020; Figure 2). In contrast, the original substrates for CRBN during development and any endogenous ligands have not yet been clarified.

SALL4 is a member of the *spalt*-like family of C2H2 zinc finger transcription factors that is involved in embryonic development. Mutations in *SALL4* are associated with Duane-radial ray syndrome/Okihiro syndrome and SALL4-related Holt-Oram syndrome, both of which show phenotypes similar to those of thalidomide embryopathy (Al-Baradie et al., 2002; Kohlhase et al., 2002, 2003). Consistent with these observations, thalidomide was found to induce SALL4 degradation in a species-specific manner (Donovan et al., 2018). *SALL4* is expressed in early embryos, including the inner cell mass, heart, and neuroectoderm. Thus, *SALL4*-deficient mice are embryonic lethal (Sakaki-Yumoto et al., 2006). *SALL4* haploinsufficiency results in death *in utero*, anorectal and heart anomalies, and exencephaly in mice. Therefore, SALL4 may be involved in thalidomide-induced miscarriage or other birth defects in humans. *Sall4* is also required for pectoral fin outgrowth (Harvey and Logan, 2006) that seems consistent with thalidomide-induced limb defects in zebrafish (Ito et al., 2010; Asatsuma-Okumura et al., 2019). However, zebrafish SALL4 is resistant to thalidomide-induced degradation (Donovan et al., 2018), suggesting the presence of other neosubstrates.

We identified p63 as another thalidomide-dependent CRBN neosubstrate and found that it was involved in teratogenic effects in the limb and ear (Asatsuma-Okumura et al., 2019). P63 is a member of the p53 transcription factor family, also known as tumor suppressors, and plays an important role in limb development (Yang et al., 2002). P63-deficient mouse embryos show severe limb defects similar to thalidomide-induced amelia and defects in craniofacial and epithelial development, suggesting that p63 is essential for ectodermal differentiation, epithelial development, and morphogenesis (Mills et al., 1999; Yang et al., 1999). In humans, mutations in *TP63* cause ectodermal dysplasia, cleft lip/palate syndrome, and congenital limb malformations (Rinne et al., 2006). Consistently, knockdown of a p63 isoform, ΔNp63, showed disruptions in epidermal growth and limb development in zebrafish (Lee and Kimelman, 2002). The similarity of these phenotypes to thalidomide embryopathy prompted us to explore the possibility of p63 as a thalidomide-dependent neosubstrate. Using zebrafish, we demonstrated that p63 isoforms ΔNp63α and TAp63α were responsible for teratogenicity in the limb and ear through thalidomide-dependent degradation, respectively (Asatsuma-Okumura et al., 2019, 2020). P63 is also expressed in the embryonic and adult mouse and human telencephalon (Hernandez-Acosta et al., 2011). Genetic p63 knockdown in the embryonic telencephalon causes embryonic cortical precursor cell apoptosis that is rescued by ΔNp63 expression (Dugani et al., 2009). Nevertheless, constitutive p63 ablation results in no deficits in neural development (Holembowski et al., 2011). In contrast, inducible p63 ablation from embryonic day 12 leads to an increase in neural precursor cell apoptosis in the embryonic cortex (Cancino et al., 2015). These observations suggest that p63 plays an important pro-survival role in the developing brain (Joseph and Hermanson, 2010), also implying that p63 may be a potential neosubstrate for thalidomide teratogenicity in the brain.

## Therapeutic Effects of Thalidomide in Humans

The effectiveness of thalidomide against multiple myeloma encouraged Celgene Corporation to develop its derivatives, lenalidomide and pomalidomide that have higher immunomodulation activities (Bartlett et al., 2004). Thalidomide and its derivatives were therefore referred to IMiDs. Consistent with their immunomodulatory activity, we demonstrated that lenalidomide and pomalidomide bounded more strongly to CRBN than to thalidomide (Lopez-Girona et al., 2012), indicating that CRBN is required for both their teratogenic and therapeutic effects. Because CRBN is a CRL4^CRBN^ substrate receptor, the substrates responsible for the therapeutic effects were explored; different ligand-dependent substrates (neosubstrates) have been identified for the therapeutic effects of thalidomide and its derivatives (Kronke et al., 2014, 2015; Lu et al., 2014; Matyskiela et al., 2016; Ito and Handa, 2020; Figure 2).

The transcription factors Ikaros (IKZF1) and Aiolos (IKZF3) that belong to the Ikaros zinc finger family (IKZF), were first identified as lenalidomide-dependent CRL4^CRBN^ substrates in multiple myeloma cell lines (Kronke et al., 2014; Lu et al., 2014). Ikaros and Aiolos degradation is the main mediator of the anti-myeloma effects of lenalidomide. We revealed that lenalidomide and pomalidomide also induced Ikaros and Aiolos degradation in T cells (Gandhi et al., 2014).

Casein kinase 1A1 (CK1α) was identified as another lenalidomide-dependent CRL4^CRBN^ neosubstrate responsible for the therapeutic effect of lenalidomide against myelodysplastic syndrome with chromosome 5q deletion (Kronke et al., 2015). CK1α is a serine/threonine kinase that plays important roles in embryonic and tumor development. CK1α inhibits p53 and negatively regulates Wnt signaling (Huart et al., 2009; Elyada et al., 2011; Wu et al., 2012). Consistently, homozygous deletion of the CK1α gene, *Csnk1a1*, in hematopoietic cells results in apoptosis through p53 activation in conditional knockout mice (Schneider et al., 2014). CK1α degradation by lenalidomide was substantially more extensive than that by thalidomide or pomalidomide, indicating that substrate recognition by CRBN differs depending on the ligand structure (Kronke et al., 2015).

In a thalidomide derivative library developed by Celgene, CC-885 was discovered to possess remarkable therapeutic effects against acute myelogenous leukemia. We identified a CC-885-dependent neosubstrate, G1-to-S phase transition 1 (GSPT1), by immuno-affinity purification and found that CC-885 has an anti-proliferative effect by degrading this neosubstrate (Matyskiela et al., 2016). Because the effects of CC-885 were beyond the scope of immunomodulatory drugs, thalidomide and its derivatives IMiDs have been collectively termed CELMoDs. This recent progress in elucidating the molecular function of CELMoDs supports the hypothesis that these small-molecule compounds confer neosubstrates on CRBN by altering substrate recognition (Chamberlain and Cathers, 2019; Ito and Handa, 2020; Figure 1).

The CRBN-based PROTAC technique utilizes thalidomide or other CRBN-binding compounds combined with small-molecule compounds that interact with the proteins of interest, allowing us to target protein degradation by recruiting CRL4^CRBN^ (Winter et al., 2015; Burslem and Crews, 2020). This targeted protein knockdown with PROTACs opens new possibilities for CELMoDs in drug discovery (Chamberlain and Hamann, 2019; Pettersson and Crews, 2019).

## NSCs in Brain Development and Neurodevelopmental Disorders

The number of NSCs is determined by the balance of proliferation, differentiation, and apoptosis. Caspase-3 and -9 knockout suppresses apoptosis, causing expansion of NSCs/radial glial cells (RGCs), leading to a larger and consequently convoluted cortical surface (Haydar et al., 1999; Rakic, 2009). NSC proliferation and differentiation are strictly regulated during brain development (Kriegstein and Alvarez-Buylla, 2009; Rakic, 2009; Aimone et al., 2014; Florio and Huttner, 2014). In the developing brain, NSCs/RGCs proliferate through symmetric cell divisions or give rise to intermediate (basal) progenitor cells or neurons by asymmetric cell division in the ventricular zone. Intermediate progenitor cells migrate basally along radial fibers and produce two neurons by symmetric cell divisions in the subventricular zone. Thus, NSCs produce differentiated cells (progenitor cells or neurons) at the expense of proliferation. Consequently, the number of NSCs/RGCs at early developmental stages impacts brain size at later stages (Golzio et al., 2012; Malhotra and Sebat, 2012). Indeed, multiple human genes associated with microcephaly, macrocephaly, and megalencephaly are involved in cell division and cell cycle regulation, such as mitotic spindle orientation, centromere formation, microtubule organization, cytokinesis, and signal transduction (Williams et al., 2008; Pirozzi et al., 2018). Moreover, microcephaly and macrocephaly are observed in neurodevelopmental disorders, autism spectrum disorder, and intellectual disability, suggesting that impaired neurogenesis in the embryonic brain accounts for susceptibility to these neurodevelopmental disorders (Courchesne et al., 2007; Vorstman et al., 2017; Bonnet-Brilhault et al., 2018).

During brain development, newly generated neurons migrate into different layers depending on the timing of their generation from RGCs (Kriegstein and Alvarez-Buylla, 2009). Early-born neurons are distributed in the deeper layers, and later-born neurons in the superficial layers; thus, cortical layers are developed in an inside-out manner. A disrupted balance between NSC/RGC proliferation and differentiation could affect cortical circuit organization, as projection neuron subtypes are determined by the cortical layers in which the neurons reside (Greig et al., 2013). Indeed, multiple susceptibility genes for neurodevelopmental disorders, such as *PTEN*, *CHD8*, and *SYNGAP1* for autism spectrum disorder; *KRAS* and *RHEB* for intellectual disability; and *DISC1*, *NRG1*, and *MAPK3* for schizophrenia are implicated in embryonic neurogenesis, including NSC/NPC proliferation and differentiation, neuron generation and migration, and post-mitotic neuron differentiation during brain development (Abrahams and Geschwind, 2008; Amaral et al., 2008; Sato et al., 2015; Vorstman et al., 2017; Sacco et al., 2018), suggesting that neurodevelopmental disorders are attributed to impaired neurogenesis in the fetal brain (Kaushik and Zarbalis, 2016; Muhle et al., 2018; Sacco et al., 2018). The pathogenic mechanisms underlying neurodevelopmental psychiatric disorders support the hypothesis that fetal development affected by *in utero* environments, including exposure to teratogens, pathogens, or maternal stress, leads to later-onset diseases (Gluckman et al., 2007).

## NSCs in Adult Neurogenesis and Mental Disorders

Fetal exposure to teratogens, including thalidomide as well as other small-molecule compounds such as valproic acid (antiepileptic), misoprostol (antiulcer drug), and ethanol, is associated with autism (Dufour-Rainfray et al., 2011). Prenatal exposure during the first trimester to antidepressants such as the selective serotonin reuptake inhibitor (SSRI), fluoxetine was associated with an increased risk of autism (Velasquez et al., 2013; Andalib et al., 2017; Millard et al., 2017). Fluoxetine promotes neurogenesis in the adult hippocampus and has been proposed to contribute to the therapeutic effects on mood disorders such as major depression in humans (Santarelli et al., 2003; Micheli et al., 2018; Planchez et al., 2020). Consistently, serotonin (5-HT) has multiple roles in adult hippocampal neurogenesis (Song et al., 2017). Most notably, it promotes NSC proliferation through the 5-HT_1__A_ receptor in the adult hippocampus (Radley and Jacobs, 2002; Banasr et al., 2004) suggesting that embryonic and adult neurogenesis share a common molecular mechanism mediated by targets of small-molecule compounds. However, adult hippocampal neurogenesis reduces dramatically with age not only in rodents (Altman and Das, 1965; Kempermann et al., 2015), but also in humans (Snyder, 2018; Sorrells et al., 2018). Therefore, reactivation of quiescent NSCs and expansion of endogenous NSCs/NPCs in the brain will be an ideal symptomatic treatment for patients with impaired adult neurogenesis resulting in neurodevelopmental disorders, mood disorders, and neurodegenerative disorders (Li et al., 2013; Herrera-Arozamena et al., 2016; Duncan and Valenzuela, 2017).

## Teratogenic Effects of Thalisomide and Potential Therapeutic Activity of CRBN in Zebrafish Brain Development

We found that thalidomide treatment during early gastrulation resulted in the generation of small heads and eyes in zebrafish embryos (Ando et al., 2019). This is consistent with thalidomide-induced birth defects in mice and humans, indicating that the mechanism underlying thalidomide teratogenicity in brain development is conserved among vertebrates (Miller and Strömland, 1999; Hallene et al., 2006; Fan et al., 2008). Knockdown of CRBN the direct target of thalidomide, elicited p53-dependent apoptosis in the presumptive brain region, suggesting that CRBN is required for neuroepithelial cell survival, including that of NSCs. This is consistent with the teratogenic effects of thalidomide in zebrafish embryos in which thalidomide induces apoptosis in the developing fin bud (Asatsuma-Okumura et al., 2019). A discrepancy in phenotypes between CRBN-knockdown zebrafish and CRBN-knockout mice, as observed for p63, remains an open question, although genetic compensation by mutant mRNA degradation was reported as the molecular mechanism underlying such discrepancies (Rossi et al., 2015; El-Brolosy et al., 2019).

CRBN overexpression by injection of mRNA into one-cell-stage embryos caused expansion of the head at later stages in zebrafish (Ando et al., 2019). CRBN overexpression caused expanded NSC marker *sox2* expression in the presumptive brain field as well as an increase in mitotic cells in the telencephalon and an increase in *her5*-positive NSCs in the midbrain-hindbrain boundary. The effects of CRBN overexpression on early brain development lead to the expanded expression of neural and glial marker genes and results in an enlarged brain. These results suggest that CRBN overexpression promotes the proliferation of *sox2*-positive NSCs in the developing brain. This conclusion is supported by the fact that another E3 ubiquitin ligase complex, CRL4^*Mahj*^, using Mahjong as a substrate receptor, promotes the exit of NSCs from quiescence and leads to the reactivation of proliferation in *Drosophila* (Ly et al., 2019). These observations may indicate that newly developed CELMoDs including thalidomide analogs corresponding to CRBN overexpression can enhance NSC proliferation if they promote stabilization by inhibition of auto-ubiquitination or degradation of endogenous substrates more efficiently or by degradation of CELMoD-dependent neosubstrates that leads to cell cycle progression or growth factor signal propagation. This type of CELMoD is a potential candidate for therapeutic drugs for treating diseases that affect adult neurogenesis.

## Conclusion

Thalidomide has multiple teratogenic effects depending on the time of exposure during development. Early exposure to thalidomide caused a small head in zebrafish embryos, consistent with an increase in the risk of autism and mild intellectual disability that often affect head size in humans. Brain size is primarily affected by the number of NSCs that is determined by the balance of growth, differentiation, and apoptosis during development. Early exposure to small molecules affecting adult neurogenesis, such as antidepressants, increases the risk of autism, suggesting a common molecular mechanism underlying NSC growth and differentiation in the developing and adult brain. Our finding that thalidomide’s direct target, CRBN, regulates NSC proliferation in zebrafish embryonic brain implies that small-molecule compounds, including CELMoDs, can promote NSC growth in the adult brain and thus might be developed as effective therapeutic drugs for regenerative medicine.

## Author Contributions

TS wrote this article. TS and HH contributed to the conceptualization. TI and HH contributed to the review and editing of this article. All authors approved the study for publication.

## Conflict of Interest

HH has received research support from Celgene/Bristol Myers Squibb. The remaining authors declare that the research was conducted in the absence of any commercial or financial relationships that could be construed as a potential conflict of interest.
